# The Impact of Built Environment on Diabetic Patients: The Case of Eastern Province, Kingdom of Saudi Arabia

**DOI:** 10.5539/gjhs.v4n4p126

**Published:** 2012-06-13

**Authors:** Bhzad Sidawi, Mohamed Taha Ali Al-Hariri

**Affiliations:** 1 Department of Architecture, College of Architecture and Planning, Saudi Arabia; 2 Department of Physiology, College of Medicine, University of Dammam, Dammam, Kingdom of Saudi Arabia

**Keywords:** diabetes, urban pollution, lifestyle, sick building syndrome, smart tools, built environment

## Abstract

At present, Diabetes mellitus is considered as one of the main threats to the human health in the 21st century. It may lead to severe conditions such as blindness, end-stage of renal disease, limb amputation and a variety of debilitating neuropathies. Previous researches indicated that diabetes is caused by a complex interaction of patient’s genetics, life-style and environmental factors. They also highlighted that providing quality and healthy built environment to citizens including diabetic patients would prevent poor and unhealthy condition. The Kingdom of Saudi Arabia (KSA) is one of top ten countries in the prevalence of diabetes. Little researches though were conducted in KSA in regards to the effect of environmental conditions of the built environment. In 2011, the present researchers have carried out a pilot survey on a number of diabetic patients to find out the possible impact of built environment settings on the patient’s lifestyle. The research explored whether diabetic patients use smart tools in their daily life to overcome the daily life’s difficulties and perform their life as normal as possible. The results showed a close link between a poor home and environmental settings, the patient’s lifestyle, and the patient’s health status. It also highlighted the absence of smart tools and systems use. The paper argues that certain changes to the built environment must be done and to provide a healthy and safe environment for diabetic patients. This would help these patients to abandon their bad habits and adopt healthier lifestyle.

## 1. Introduction

Diabetes is one of the most serious common non-communicable diseases that faces people worldwide and diabetic people are patients whose life relies on continuous support, care and monitoring. Researchers highlighted that diabetes is caused by a complex interaction of patient’s genetics, life-style and environmental factors (see for instance Stewart et al., 2011). The bio-psychosocial model as a new paradigm recognizes that disease and behavior are functions and result from the interaction among biological, psychosocial, developmental, socio-cultural and ecological factors (Madhu & Sridhar, 2001). Urban sprawl has been linked to a variety of health-related concerns including air pollution, water quality, traffic accidents, and mental health issues (Bray, Vakil, & Elliott, 2005). The study indicates that urban design settings and various aspects of the built environment can also play an important role in the development of diabetes T2DM and its risk factors (Allanah, Ashley, & Farley, 2010). The sick built environment may also enforce people to adopt habits and lifestyle that is unhealthy and complicate their health status. Studies of the built environment acknowledge that aspects of our physical surroundings can shape choices about diet and physical activity – both important contributors to the development of diabetes (ibid).

So the built environment should be properly designed to create a healthy environment and lifestyle. This includes the study of physical surroundings’ features such as proximity of grocery stores, safe and pleasant opportunities for physical activity, and time spent commuting. Changes should be made to the built environment to positively influence health outcomes and to be made more activity-friendly by improving sidewalks and bicycle paths, building recreation spaces, and instituting mixed land-use patterns in more suburban areas to provide better walking destinations (ibid). Smart tools would help people and especially with some type of disability to overcome the life difficulties and facilitate their access to places within the built environment. This paper focuses on the built environment and lifestyle factors that would contribute to the development of diabetes and discusses possible solutions that improve the health status of diabetic patients.

## 2. The Diabetes Types and Complications

Diabetes mellitus (DM) is defined by American Diabetic Association (ADA) and adopted by World Health Organization (WHO) as the following: an individual is said to have normal blood glucose when fasting plasma glucose (FPG) is < 6.1mmol/L (110 mg/dL), impaired fasting glucose (IFG) when FPG is between 6.1-6.9 mmol/L (110 and 125 mg/dL), and DM when FPG is ≥ 7.0mmol/L (126 mg/dL) or a random value at or above 11.1mmol/L (200 mg/dL) (Alberti & Zimmet, 1998). There are – in general- two types of diabetes. In Diabetes type I (T1DM), the person’s own body has destroyed the insulin-producing beta cells in the pancreas. Although type II diabetes mellitus (T2DM) can be caused by genetic factors, unhealthy lifestyle happens to be the main cause. A person with T2DM has one of two problems, and sometimes both: a) not enough insulin is being produced; and b) the insulin is not working properly. Diabetes mellitus is the most common non-communicable disease worldwide and the fourth to fifth leading cause of death in developed countries. The International Diabetes Federation (IDF) indicates that the prevalence of diabetes mellitus has reached epidemic levels globally. Estimates for 2010 indicate that 285 million adults have diabetes in the seven regions of the IDF. These numbers represent an increase of 39 million from 2007 and an expected continued increase to 439 million in 2030 (International Diabetes Federation, 2009). While the direct symptoms of diabetes, such as thirst, frequent urination and fatigue, can be mild and may cause little interruption to activities of daily living, it is the complications of the disease, including blindness in adults (Jeppesen & Bek, 2004) non-traumatic lower-limb amputation (Chaturvedi, Stevens, Fuller, Lee, & Lu, 2001) and kidney failure resulting in transplantation and dialysis (Atkins, 2005). In addition, the risk of coronary heart disease is two to four times higher in diabetic patients. The risk of stroke or peripheral vascular disease also increases strongly. In fact, the management and treatment of diabetes mellitus mainly T2DM is can considered more than the mere control of blood glucose values, asking for a multidisciplinary approach (i.e. shared care) to reduce macro- and micro-vascular risk factors (American Diabetes Association, 2006).

### 2.1 Diabetes and Socio-economic Factors

Diabetes mainly T2DM is a lifestyle disorder, and many studies indicates that, the incidence of this disease is projected to increase as populations age (International Diabetes Federation, 2007), urbanization increases (Bray, Vakil, & Elliott, 2005), diets become ‘westernized’ (Schulze, Manson, Willett, & Hu, 2003), and levels of physical activity decrease (Eckel et al., 2004). Passive entertainment exemplified by television viewing and computer games along with intake of meals; all contribute to disorders of lifestyle (Peckhan, 1998, Ebbeling, Pawlak, & Ludwig, 2002). This imbalance between energy intake (i.e. feeding) and energy expenditure (i.e. physical activity) unfortunately leads to obesity (Michaud et al., 2001, Ebbeling, Pawlak, & Ludwig, 2002). Moreover, a growing body of evidence suggests an association between diabetes with socioeconomic and built environment conditions (Stewart et al., 2011).

Previous surveys from KSA suggested that diabetes is present in epidemic proportions throughout the country with exceedingly high rates concentrated in urban areas (Alzaid, 1997). A Study showed that, prevalence of diabetes mellitus is highest among the Northern Saudi population (Al-Nozha, Al-Maatouq, & Al-Mazrou, 2004). A US study showed that, incidence of diabetes was higher among African-American women (i.e. black women) in low socioeconomic status (SES) versus higher SES neighborhoods (Krishnan, Cozier, Rosenberg, & Palmer, 2010), greater risk of coronary heart disease in socioeconomically disadvantaged versus more affluent census block groups (Diex Roux et al., 2001), and higher rates of obesity in socioeconomically deprived neighborhoods compared to more affluent neighborhoods (Cubbin et al., 2006). Other investigations indicate higher rates of diabetes (Nemmar et al., 2002; Lockwood, 2002) and obesity (Nemmar et al., 2002; Sun el al., 2009; Kelishadi, Mirghaffari, Poursafa, & Gidding, 2009) in rural areas relative to urban centers. Poorer health status in socioeconomically deprived and rural environments may reflect, in part, the inaccessibility of such built environmental features as public pools, recreation centers, physical fitness facilities, parks, sidewalks, and streetlights (Goldberg et al., 2000). Stewart et al (2011) conducted a study in the USA that explores potential county-level associations between diabetes prevalence among adult African Americans and five measures of the socioeconomic and built environment—persistent poverty, unemployment, rurality, number of fast food restaurants per capita, and number of convenience stores per capita. They found Diabetes prevalence rates in South Carolina are among the highest in the nation and there is association between the socioeconomic measures and diabetes (Stewart et al., 2011).

### 2.2 Environmental Pollution and Diabetes

#### 2.2.1 Urban Pollution

Diabetes is influenced not only by factors in each individual, but by the environment that surrounds the individual. There are many causes to Urban Contamination such as exposure to a chemical or other toxic substance originating from a source outside the building, e.g. motor vehicle exhaust fumes, construction activity, underground petrol spillage (Melius, Wallingford, Keelyside, & Carpenter, 1984). One important regulated pollutant is particles equal or less than 10 microm in diameter (PM10), formed as a result of fossil-fuel combustion by motor vehicles and stationary sources such as power plants. PM10 are generated from combustion emissions such as automobile exhaust or wood or coal burning and industrial emissions from smelters, paper and steel mills, or cement plants. PM10 can deposit in the lower airways changing portions of the lung, even reaching the circulatory system and, therefore, are considered to be of greater health significance (Nemmar et al., 2002). Many researchers have found association between environmental pollution and the pathogenesis of diabetes (Papazafiropoulou, Kardara, & Pappas, 2011) and especially with those considered as a sensitive population such as children and with cardiovascular diseases. Balfour and Kaplan (2002) reported that poor lighting, excessive noise, heavy traffic, and lack of public transit are associated with loss of physical function in adults over 55 years of age. They suggested that these detrimental environmental features discourage neighborhood excursions. In addition, individuals with diabetes are at greater risk of dying (Goldberg et al., 2001) and being hospitalized for heart disease during periods of high environmental pollution (Zanobetti & Schwartz, 2002). This phenomenon has been explained partially due to the association between exposure to environmental pollution and markers of cardiovascular risk related to decreased heart rate variability (Creason et al., 2001; Tunnicliffe, Hilton, Harrison, & Ayres, 2001) and increased levels of thrombotic and inflammatory factors (Donaldson, Stone, Seaton, & MacNee, 2001; Peters et al., 2001) since inflammation is the key pathway leading to atherosclerosis and subsequent adverse cardiovascular events. The diabetic subjects with cardiovascular diseases are more susceptible to the detrimental effects of environmental pollution than diabetics without cardiovascular diseases (Goldberg et al., 2006; Filho et al., 2008). Other studies in children have showed that environmental pollution is linked with the development of T1DM (Hathout, Beeson, Ischander, Rao, & Mace, 2006).

A lot of recent studies have showed a relationship between PM10 and diabetes mellitus (Lockwood 2002; Sun et al., 2009; Kelishadi, Mirghaffari, Poursafa, & Gidding, 2009). The long-term exposure to environmental pollution particles, 2.5 mm in aerodynamic diameter (PM2.5) has been found to be associated with a higher relative risk of mortality among people with diabetes compared with the general population (Jerrett et al., 2005). Also similar association found between prevalence of T2DM and PM2.5 (Pearson, Bachireddy, Shyamprasad, Goldfine, & Brownstein, 2010). A study by Brook et al., (Brook, Jerrett, Brook, Bard, & Finkelstein, 2008) investigates the relation between diabetes and traffic-related NO^2^ in two different cities; Toronto and Hamilton. They found significant association between the diabetes prevalence and exposure to traffic-related NO^2^. Lee et al (Lee et al., 2006) have found similar association between the organic pollutants and prevalence of T2DM (see similar findings by Ukropec et al., 2010). Hathout et al. (2002) found association between Prevalence of T1DM and substances O^3^, SO^2^ and substances NO^2^, SO^2^ and SO^4^. This possible association between environmental pollution and increased incidence of diabetes should motivate policy makers to issue prevention policies to reduce air pollution and therefore, its, harmful consequences.

#### 2.2.2 Sick Building Syndrome

Buildings are complex environments which can trap and concentrate pollutants as well as generate them. Outside pollutants find their way into buildings through air intakes and inadequate filtering systems. As long as ample ventilation ensures a constant supply of fresh air, indoor pollution problems may be kept to a minimum. However, general ventilation is often inadequate and office equipment may have no local exhaust system venting fumes to the outside (London Hazards Centre trust, 1990). Youle (1986) has found that air-conditioning systems giving rise to symptoms of sick building syndrome. Researchers have found association between the ventilation rates, CO^2^-concentrations with health problems in commercial and institutional (Seppänen, Fisk, & Mendell, 1999) and Office Buildings (Apte, Fisk, & Daisey, 2000). Sick building syndrome has a number of health symptoms such as: lethargy and tiredness, headache, dry blocked nose, sore dry eyes, sore throat, dry skin and/or skin rashes, allergy etc (World Health Organization, 1983; Morris & Hawkins, 1987; Wilson & Hedge, 1987). Allergic reaction in sensitive individuals was associated with chest tightness, difficulty in breathing, fever and headache. These health problems may aggravate diabetes. Certain materials are identified as causes of Contamination inside the buildings: exposure to chemical or other toxic agent generated within the office space, e.g. methyl alcohol from spirit duplicator, methacrylate from a copier, sulphur dioxide from a heating system, amines used in a humidification system, chlordane used as a pesticide (Melius et al., 1984).

### 2.3 Potential Solutions

The green and sustainable buildings design aim is to design buildings that reduce the overall impact of the built environment on human health and the natural environment by: efficiently using energy, water, and other resources, protecting occupant health and improving employee productivity, and reducing waste, pollution and environmental degradation. These buildings theoretically use zero energy and do not release harmful substances to the environment, enrich the environment in a way or another thus it mimics the nature. Such buildings should provide healthy indoor environment to its inhabitants and expose them to adequate amount of natural light and ventilation, views of greenery, and close proximity to outdoor green space. Landscape architecture appears to be the primary key at the finest scale to sound mind and body and simply viewing nature reduces the stress of daily urban life (Ulrich, 1979; Jackson, 2011). Jackson and Kochtitzky (2001) advocate providing neighborhood opportunities for walking to accomplish routine activities such as shopping and going to work. Andersen et al., (1999) report that lifestyle activities such as structured aerobic exercise are effective in losing weight. Critical to this strategy is conducive neighborhood design. Physical activity is defined as the total of planned and repetitive movements of skeletal muscles, which are performed using energy. The beneficial effects of exercise in patients with T2DM have been recognized long time ago. Today, the beneficial role of exercise has been fully documented and exercise should be incorporated systematically in the treatment of patients with diabetes (Lecomte, Romon, Fosse, Simon, & Fagot-Campagna, 2008; De Feo et al., 2006; Sato et al., 2007; La Monte et al., 2005). Moreover, exercise has a significant role in the regulation of blood glucose, improves insulin action, metabolism of proteins and fats, prevents complications of diabetes, improves muscle flexibility and strength, has beneficial effects on the cardiovascular system and increases life expectancy of the patients. In addition, physical activity is beneficial for the mental state of the individual, because it increases the energy of the human body, improves self-esteem and decreases depression (Lecomte et al., 2008; De Feo et al., 2006; Sato et al., 2007; Colberg, 2007). It is widely accepted that healthy nutrition is the basis for the treatment of T2DM. It contributes positively to the maintenance of blood glucose within normal range and minimizes the complications of the disease. Unfortunately the diet in urban dwellers are usually contains a greater proportion of refined carbohydrates, less fiber and more fat in comparison to that in rural environment (Stephen & John, 1997).

Smart technology is defined as the technology used to make all electronic devices in a building act “smart” or more automated. The smart technology aim is to help people including those who have special needs i.e. disability to control the environment that is around them. Also, it helps them to get support and assistance from the surrounding environment. A smart home is a home that equipped with special structured wiring and devices that enable occupants to remotely control or program an array of automated home electronic devices by entering a single command (SearchSMB.com, 2007). Smart products and services can be divided into six categories (Roe, 2007) namely: comfort, energy management, multimedia, and entertainment, healthcare (European Senior Watch Observatory and Inventory, 2002), security, safety and communication. Kanstrup et al. (2008) reviewed the ICT designed for diabetics. They categorized it into the following categories: medical devices, self-management tools, education and Games, and websites and social networks. Recently, home tele-monitoring has been applied as a new approach in the management of diabetes mellitus, to generate a registry of diabetic patients and provide tools for patient tracking and follow-up, in which physiological and biological data are transferred from the patients’ home to the tele-monitoring centre by giving physician access to monitor patient’s information, enabling identification of at risk population interpret the data, and providing clinical decisions (Roine, Ohinmaa, & Hailey, 2001). However, home tele-monitoring is an integral part of a broader view of deinstitutionalization and reflects a societal orientation toward maintaining patients in their homes (Canadian Home Care Association, 2008). Many studies concluded that home tele-monitoring is as effective in glycemic control as the traditional approach to home follow-up (Paré, Jaana, & Sicotte, 2007).

## 3. The Research Objectives and Methodology

The research has a set of objectives and these are:

- To find out the relation between the frequency and progress of diabetes symptoms, the patient’s lifestyle, and built environment settings
- To make recommendations on how to enhance the built environment to be healthy, supportive and assistive

To achieve the research objectives, a questionnaire survey was carried out to assess the patients’ views about the potential impact of built environment on the progress of the disease. Necessary permission was obtained as the protocol was reviewed and approved by the medical ethical committee of the University of Dammam. The field survey was used to target 36 patients who visit diabetes’ clinic, King Fahid Teaching Hospital of the University of Dammam, in March/April 2011. A questionnaire was prepared to inspect the diabetic patients’ views about their environment and lifestyle. Patients were randomly chosen. The choice was based on the following criteria: the exclusion of severely ill, children, and women patients as interviews with women need special arrangements; and the inclusion of T1& 2DM male adults, age 18-60 years old. During the appointment with each diabetic patient at diabetes’ clinic, King Fahid Teaching Hospital of the University of Damamm, the patient was asked to fill in the questionnaire. Thirty three male patients filled in the questionnaire and three returned invalid questionnaires. The sample size however, (i.e. number of respondents) was small and the following simple statistical tests were applied: Mean, percentage, Chi-square for the test of significance, and Cramer’s test of correlation. It should be mentioned that the study reports the significant results only (i.e. 0.5> P).

## 4. The Field Survey Results

The validity and reliability of the data was tested. The reliability (i.e. Cronbach Alpha) and validity values were 0.947 and 0.897. Patients are from Dammam and Al Khober cities and few are from AlAhsa city and Al Qateef city. The analysis of the questionnaire results showed that 21% of the respondents are between 20 to 40 years old, 55% are between 41-60 years old and the rest are between 61-80 years old. Around one third of the respondents use insulin and tablets. Around 50% use tablets and less than a quarter use insulin. Around one third of the respondents have high last Fasting blood Glucose reading i.e. more than 200 mg/dL. And one third has medium reading i.e. 151-200 mg/dL and the rest have low reading. Around 21% of the respondents said the Onset date of the disease is from one to five years, 42% said it is from 6-10 years ago, 12% said that they have it from 11 to 15 years whereas 21% said they have more than 15 years ago. 91% of the respondents have lived in their present address more than 5 years and 6% have been less than 5 years whereas the rest did not reveal the period of residence. 24% have experienced accidents since the onset of the disease and 73% have not. 30% said that the highway is less than 500 meters from their homes, whereas 80% of the respondents said that the main road is less than 500 meters from their homes. 41% of the respondents pointed out that the public amenities are less than 200 meters and 44% said it is less than 500 meters. Around one third said the public gardens are less than 500 meters. However, 42% of the participants said it is more than 2000 meters from home. 41% said that the Recreation and sport Centre is more than 2000 meters from home, whereas around 22% said it is less than 500 meters from home.

**Figure 1:**
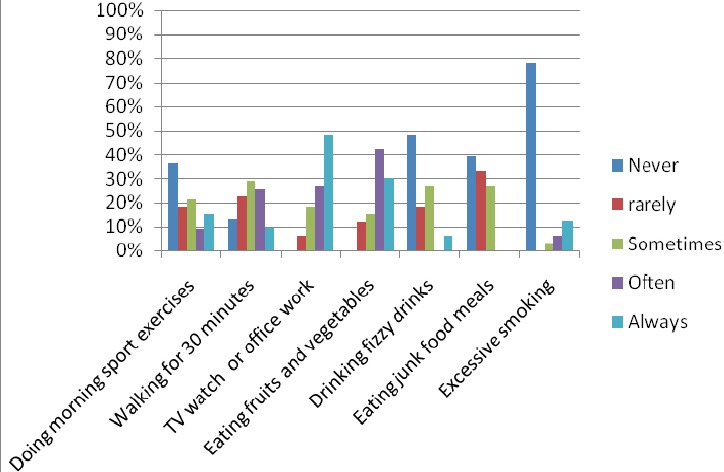
The frequency of daily lifestyle habits

More than half of the participants said that they have never or rarely done any morning exercises on daily basis (Table 1 and Figure 1). Only a quarter of the respondents have done frequently/always morning exercises. More than one third of the surveyed patients said they never/rarely walk for 30 minutes every day. Around the quarter said they did it frequently and only 10% said they always do it. Around 75% of the respondents said they often/always watch TV or carry out office work on daily basis. 72% of the respondents said they often/always eat fruits and vegetables every day. 27% of the respondents said they sometimes drink fizzy drinks and eat junk food every day. Around 20% said they smoke excessively on daily basis. Since they have lived in their homes, the respondents complained mostly from the following environmental issues: noise from the traffic, little sun penetration to their homes, noise from neighbors, difficulty to wonder around within the neighborhood, the lack of cleanness of the neighborhood (Table 2).

**Table 1:** The frequency of daily lifestyle activities of the patient since the onset of diabetes

Daily lifestyle activity	Never	rarely	Sometimes	Often	Always
Doing morning sport exercises	36%	18%	21%	9%	15%
Walking for 30 minutes	13%	23%	29%	26%	10%
TV watch or office work	0%	6%	18%	27%	48%
Eating fruits and vegetables	0%	12%	15%	42%	30%
Drinking fizzy drinks	48%	18%	27%	0%	6%
Eating junk food meals	39%	33%	27%	0%	0%
Excessive smoking	78%	0%	3%	6%	13%

Sample size = 30

**Table 2:** How often a number of conditions are experienced by the patient since he has resided in his/her present address

Home and neighborhood environment’s condition	Never	rarely	Sometimes	Often	Always
Too little air/ventilation	70%	3%	18%	6%	3%
Annoying air draft	69%	9%	13%	3%	6%
Hot air conditions	58%	18%	18%	3%	3%
Little sun penetration	55%	6%	15%	18%	6%
Poor air quality	72%	13%	9%	6%	0%
Unpleasant outside views	91%	3%	3%	0%	3%
Unpleasant odours	58%	15%	24%	0%	3%
The lack of cleanness in the neighborhood	52%	12%	30%	6%	0%
The quality of home finishing	69%	6%	16%	0%	9%
The uncomfortable home organization and small size	61%	15%	9%	3%	12%
The uncomfortable home furniture	73%	9%	15%	0%	3%
Noise from the traffic	39%	10%	23%	16%	13%
Noise from neighbors	53%	6%	31%	3%	6%
Pollutant neighborhood	61%	16%	23%	0%	0%
Difficulty to wonder around within the neighborhood	56%	9%	22%	3%	9%

Sample size= 30

The least complains were about the followings: the uncomfortable home furniture, poor air quality in the house, and unpleasant outside views (Table 2). Around one third said they always/often suffer from the following health problems since they have had the diabetes: Paresthesia, blurred vision, extreme tiredness, and extra fat problems since the onset of the diseases (Table 3). 44% of the respondents said they suffer always/often from blood pressure problems and 19% experience drowsiness since they have the diabetes. However, around half of them said that they sometimes have stress and around one third have extreme tiredness. Few of the respondents said they experience loss of sensation particularly in the foot limbs or Cardiatric problems (Table 3). Only one third of the respondents use Remote A/C and heating control and tele-services through the Internet and very few have remote lighting control (Table 4). Nearly all the respondents do not have: electronic medical devices and medical aids, remote lighting control, life safety System, virtual clinic/hospital tools, electronic Security and anti-burglary system and individual wellness monitoring tools.

**Table 3:** How often the patient has experienced a number of health problems since the onset of diabetes

Type of symptoms and health problems	Never	rarely	Sometimes	Often	Always
Paresthesia	28%	13%	25%	9%	25%
Blurred vision	27%	18%	18%	18%	18%
Extreme tiredness	12%	12%	36%	15%	24%
Stress	9%	21%	48%	6%	15%
Loss of sensation particularly in the foot limbs	65%	16%	10%	3%	6%
Cardiatric problems	75%	9%	6%	0%	9%
Blood pressure problems	41%	3%	13%	19%	25%
Extra fat problems	36%	12%	12%	12%	27%
Drowsiness	34%	19%	28%	13%	6%

Sample size= 30

**Table 4:** How frequent the patient has used smart home systems

Type of smart home systems	Do not have	Never used	Rarely used	Sometimes used	Often used	Always used
Electronic medical Devices and medical aids	100%	0%	0%	0%	0%	0%
Remote A/C and heating control	58%	0%	6%	9%	6%	21%
Remote lighting control	88%	0%	0%	6%	3%	3%
Life safety System	91%	0%	3%	0%	3%	3%
Virtual clinic/hospital tools	97%	0%	0%	3%	0%	0%
Electronic Security and anti-burglary system	94%	3%	3%	0%	0%	0%
Individual wellness monitoring tools	76%	0%	0%	0%	9%	15%
Tele-services through the Internet	36%	0%	21%	12%	9%	21%

Sample size= 30

In regards to cross tabulation results, only significant links between variables (i.e. 0.05 > P) are reported here. The examination of data shows that the older patients drink less fizzy drinks than younger patients and this can be considered as a healthy phenomenon (P = 0.011). The older patients seem to be more sensitive to the environmental conditions and complain more about the pollutant neighborhood than the younger patients (P = 0.020). Also, they suffer Extreme tiredness and loss of sensation particularly in the foot limbs more than younger patients (P = 0.005). Patients with lower last fasting blood Glucose reading, walk more frequently than patients with higher Glucose reading (P = 0.013). Al khober and Dammam’s patients tend to walk more frequently than AL Ahsaa and Al Qateef patients (P = 0.023). However, they watch more TV or carry out office work than Al Ahsaa and Al Qateef patients (P = 0.033). Al Qateef patients drink more frequently fizzy drinks (P = 0.003) and do excessive smoking more than other patients (P= 0.026). However, as the sample of Al Qateef is small, the results cannot be generalized to the whole population. Al Qateef patients have suffered more frequently than other patients from the following sick indoor conditions: annoying air draft, hot air conditions, poor air quality, unpleasant odours and extreme tiredness (P = 0.041, P = 0.0002, P = 0.00004, P = 0.023 and P = 0.039). Patients who tend to watch more frequently the TV or carry on office work are those who live in an area whereas the recreation and sport Centre is farther (P = 0.015). Patients who experience more frequently the loss of sensation particularly in the foot limbs, said that they suffer more frequently from too little indoor air/ventilation, annoying air draft and noise from neighbors (P = 0.001, P = 0.0001 and P = 0.007). Patients, who experience more frequently from blood pressure problems, said they suffer more frequently from neighbors’ noise (P = 0.012) and those who experience more frequently from drowsiness said that they have higher difficulty in wandering around within the neighborhood (P = 0.038).

## 5. Discussion and Conclusion

Results of the present study supported with the literature review showed the negative possible impact of the existing built environment conditions in the Eastern province, KSA on the diabetic patients. The survey Results showed that public gardens and recreation and sport Centers are not well located within the urban context. This would make it difficult for the patients to enjoy walking to and within these centers and parks particularly during the harsh hot weather that last around 6 month in KSA. Similar findings were found by Allanah, Ashley, & Farley (2010) who stated that the ill designed urban settings would enforce people to adopt unhealthy lifestyle thus contribute to the development of T2DM. Therefore, it is no surprise to find that patients who tend to watch more frequently the TV or carry on office work are those who live in an area whereas the recreation and sport Centre is far from their homes. In respect to the lifestyle activities, more than half of the participants said that they have never or rarely done any morning exercises and more than one third said they never/rarely walk for 30 minutes on daily basis. Around three quarters of the respondents often/always watch TV or carry out office work every day meanwhile around the same percentages often/always eat fruits and vegetables on daily basis. Around one third said they always/often suffer a number of health problems. Eckel et al. (2004), Pekhan (1998) and Michaud et al (2001) have linked the passive entertainment, lack of physical activities and bad eating habits with the increasing incidence of the diabetes. The respondents complained mostly from a number of environmental issues such as noise from the traffic and neighbors, little sun penetration to their homes, difficulty to wonder around within the neighborhood, and the lack of cleanness of the neighborhood. The results showed that the frequency of the symptoms is higher for patients who suffer more frequently from indoor/outdoor environmental/urban conditions. The older patients seem to be more sensitive to the environmental conditions than the younger patients. Papazafiropoulou, Kardara, & Pappas (2011); and Balfour and Kaplan (2002), found an association between the environmental pollution and the pathogenesis of diabetes, particularly among old people and these who have cardiovascular diseases (see also Goldberg et al., 2006; Filho et al., 2008). Patients who experience more frequently from drowsiness said that they have higher difficulty in wondering around within the neighborhood. This clearly indicates that the present built environment in the Eastern province, KSA is not designed to be friendly and assistive to users. Previous research has generally points out that poorly designed urban settings would discourage neighborhood excursions (see for instance Goldberg et al., 2000)

The previous research demonstrated the usefulness of smart technology for diabetic patients (see for instance Roine, Ohinmaa, & Hailey, 2001), though this research found that it is hardly used by the participating patient. The researchers would like to point out the importance of providing healthy built environment to citizens. However, this built environment whether it is indoor or outdoor should be supported and equipped with smart tools that assist diabetic patients in their daily life. The researchers appreciate the limitation of the research but argue that it can be used as a foundation for further research. Future research should explore how a healthy environment should be designed and implemented taking into account the local environmental, cultural and lifestyle issues in KSA and Eastern Province in particular. It also may explore the impact of implementing smart tools on Urban and home levels on diabetic patients’ life and interaction with the built environment.

## Appendix

**Table:**

Reference	Sample type	Sample size	Place
Al-Nozha, Al-Maatouq, & Al-Mazrou, 2004	Saudi subjects in the age group of 30-70-years of selected households over a 5-year period between 1995 and 2000	16917 participants	KSA
Atkins, 2005	Adults	N=11247	Victoria, Australia
Allanah, Ashley, & Farley, 2010	Review of previous studies	-	Canada
Balfour and Kaplan, 2002	participants in the Alameda County Study who were aged 55 years and older and functionally healthy	883	Alameda County, USA
Bray, Vakil, & Elliott, 2005	Literature review	-	Canada
Chaturvedi, Stevens, Fuller, Lee, & Lu, 2001	Men and women aged 35 to 55 years	N=3443	American Indian, Cuban, European and East Asian WHO centers
Creason et al., 2001	Elderly (mean age 82) nonsmoking residents	N=56	Retirement center in Baltimore County, Maryland, USA
Cubbin et al., 2006	Women and men aged over 20 living in 434 neighborhoods	N= 5883	Taiwan
De Feo et al., 2006	Literature review		
Diex Roux et al., 2001	Low and high income persons	N=13009 participants	USA
Donaldson, Stone, Seaton, & MacNee, 2001	Elderly individuals	-	UK
Eckel et al., 2004	Executive Summary	Varied	varied
Filho et al., 2008	diabetic and non-diabetic patients who have cardiovascular diseases	N= 45000 cardiovascular emergency room visits	São Paulo, Brazil
Goldberg et al., 2000	residents of Montreal	N=140939	Montreal, Quebec
Goldberg et al., 2001	residents of Montreal	N=140939	Montreal, Quebec, Canada
Goldberg et al., 2006	residents of Montreal	N= - (Time series study)	Montreal, Quebec, Canada
Hathout et al, 2002	healthy children before and after 5 year of age	N=3	USA
Hathout, Beeson, Ischander, Rao, & Mace, 2006	Children	N= 402	California, USA
Jerrett et al., 2005	Respiratory clinic patients	N= 2360	Los Angeles, USA
Jeppesen, P. & Bek, T., 2004	Diabetes patients	N=7527	Århus County, Denmark
Kelishadi, Mirghaffari, Poursafa, & Gidding, 2009	children, aged 10-18 years	N=374	IRAN
Krishnan, Cozier, Rosenberg, & Palmer, 2010	African-American women, aged 30–69 years,	46382 participants	USA
Lee et al., 2006	Adult participants	N= 2016	Korea
Lecomte, Romon, Fosse, Simon, & Fagot-Campagna, 2008			
Nemmar et al.; 2002	Healthy volunteers	N= 5	Belgium
Michaud et al., 2001	Experimentation on Mice	The number of Mice is: 15 *Sim1*^+/–^males, 13 *Sim1*^+/+^males, 19 *Sim1*^+/–^females and 17 *Sim1*^+/+^females.	Canada
Papazafiropoulou, Kardara, & and Pappas, 2011	Literature review		
Peters et al., 2001	Randomly selected men aged 45 to 64 years free of cardiovascular disease	N=631	Germany
Seppänen, Fisk, & Mendell, 1999	Mixed	N= 30000 subjects	Helsinki, Finland
Stewart et al., 2011	Adults aged 18 and older enrolled in South Carolina Medicaid, between July 2008 and June 2009	N = 442830	USA
Sun el al., 2009	Male C57BL/6 mice	n=14 per group	USA
Tunnicliffe, Hilton, Harrison, & Ayres, 2001	Normal and 12 asthmatic adults	N=12	UK
Ukropec et al., 2010	Adults	N= 2047	Bratislava, Slovak Republic
Zanobetti, & Schwartz, 2002	Medicare recipients (aged_65 years) residing in Allegheny County (Pittsburgh area), Pennsylvania	N= 55019	USA
